# Christianity and Eugenics: The Place of Religion in the British Eugenics Education Society and the American Eugenics Society, *c*.1907–1940

**DOI:** 10.1093/shm/hku008

**Published:** 2014-04-08

**Authors:** Graham J. Baker

**Keywords:** Eugenics, religion, reform, Britain, USA

## Abstract

Historians have regularly acknowledged the significance of religious faith to the eugenics movement in Britain and the USA. However, much of this scholarship suggests a polarised relationship of either conflict or consensus. Where Christian believers participated in the eugenics movement this has been represented as an abandonment of ‘orthodox’ theology, and the impression has been created that eugenics was a secularising force. In contrast, this article explores the impact of religious values on two eugenics organisations: the British Eugenics Education Society, and the American Eugenics Society. It is demonstrated that concerns over religion resulted in both these organisations modifying and tempering the public work that they undertook. This act of concealing and minimising the visibly controversial aspects of eugenics is offered as an addition to the debate over ‘mainline’ versus ‘reform’ eugenics.

It is rare for studies of eugenics not to mention the question of religion. The ethical issues surrounding eugenic theories raised questions for religious believers, and this subject has featured prominently in the existing historiography. Some have used ‘religion’ as an explanatory tool for understanding the varied levels at which eugenics was accepted in different regions;^1^ others have made reference to the religious beliefs of high-profile members of the eugenics movement;^2^ and there have been detailed studies of the interaction of American religious leaders with the eugenics movement.^3^ In all cases, however, the primary focus has been upon the *response* of religious believers towards eugenics. Examples have been given of religious believers who stood on either side of the eugenics debate, resulting in a model that suggests a polarised response—one of either conflict or concession.^4^ These narratives have run in parallel with studies of the religious beliefs of scientists, such as Julian Huxley and Francis Galton, who promoted a vision of a secular religion based on the principles of evolution and eugenics. Their ideas will be discussed below, with particular emphasis on how these thoughts were interpreted by eugenics societies that sought to enjoy constructive dialogue with more traditional faith communities.

Other studies have attempted to describe the general pattern of the response of Christian groups towards eugenics. Véronique Mottier has noted that Protestant countries tended to practise eugenics more readily than Catholic ones, however she suggests that religion was not the decisive factor in determining the implementation of eugenic legislation: the balance of regional and national power in the provision of welfare was crucial.^5^ Marouf Hasian Jr., has demonstrated that eugenic theories in the USA were allied with nativist and anti-Catholic prejudice.^6^ In this case conflict was divided along religious, cultural, and racial lines; eugenics was just one more factor added to an existing split. In general, where religious believers responded positively to eugenics, these individuals have been identified as those who abandoned, or adapted, elements of ‘orthodox’ theology. Christine Rosen has argued that it was religious leaders who had ‘moved away from traditional religious tenets’ that were most supportive of eugenics. She highlighted ministers in the Social Gospel movement, ‘liberals’, and ‘modernists’, as those with the most representation in eugenics organisations; however, Rosen also notes the influence that such members were able to exert by offering criticism from within. This included advice from members such as Fr. John Cooper, a Catholic Priest.^7^ Jonathan Rose, in his study on working-class thought, has discussed the case of Ruth Slate; Rose argues that Slate became interested in the ‘sex question’ and attended lectures about eugenics after she had adopted the ‘new theology’ of Reginald John Campbell.^8^ The impression created by this scholarship is that eugenics was an aggressive force of secularisation, and religious participants were passive, liberal believers who sacrificed orthodoxy to the eugenic cause.^9^

In this article I seek to demonstrate that far from being a passive or retreating human tradition, religious ideas and cultural values associated with religion exerted a powerful influence upon eugenics organisations in Britain and the United States as they strove to make their campaigns palatable to popular society. Two organisations have been selected to illustrate this: the Eugenics Education Society based in London; and the American Eugenics Society. These organisations were both primarily concerned with the circulation of existing eugenic knowledge, rather than upon new scientific research; their attempts to engage with religious ideas reflect a broader vision to see the application of eugenic ideology and the modification of existing social mores. In examining the response from these organisations towards religious ideas it is possible to see a common drive for a discreet and sensitive promotion of eugenics, which I argue has a direct bearing upon the wider question as to the existence of ‘mainline’ and ‘reform’ eugenic ideology.

## The Eugenics Education Society

From its foundation, in 1907, the Eugenics Education Society (EES) made no secret of its ambition to orchestrate the reorientation of ethics according to biological laws. The first issue of its journal explained the objectives of the organisation as follows:

- Persistently to set forth the National Importance of Eugenics in order to modify public opinion, and create a sense of responsibility in the respect of bringing all matters pertaining to human parenthood under the domination of Eugenic Ideals.
- To spread a knowledge of the Laws of heredity so far as they are surely known, and so far as that knowledge might affect the improvement of the race.
- To further Eugenic Teaching, at home, in the schools, and elsewhere.^10^

The message was clear: public opinion was to be ‘modified’, but a new sense of ‘responsibility’ regarding parenthood had to be *created*—and one which recognised the ‘domination’ or overriding importance of eugenics. With this forceful statement of the supreme importance of eugenic values it might be reasoned that the perception of a fiercely secularised organisation holds water. However, the actions of the EES suggest otherwise. In February 1908, the society invited the Bishop of Southwark to speak at its ‘General Meeting’. When he declined, stating he was unable to investigate the organisation sufficiently enough to ‘connect himself with it publicly’, the committee turned to another Bishop. The minutes first recorded the Bishop of Stepney, before this was crossed out and the Bishop of Ripon written above. It is unclear if this amendment reflected a changed decision, a response to another refusal, or simply a mistake by the secretary, but it is very clear the EES coveted the endorsement of a Bishop for its work.^11^

The desire of the EES committee to win support from religious figures was reflected in the decisions that were made in founding its ‘quarterly journal’, *The Eugenics Review*, which it was hoped would make ‘the papers read before [EES audiences] … available to a larger public’ and ‘serve as an organ for directing popular thought on Eugenics’.^12^ The opening editorial in the first issue of the journal, published in April 1909, highlighted religion as one of a number of ideologies it need not be in conflict with:

The EUGENICS REVIEW … has a definite plan and purpose, the noblest that can be imagined—the betterment of the Human Race. … It owes no allegiance to any political party; it is neither liberal, nor conservative, nor socialist; still it is each of these in turn in the interest of the progress of humanity. Lastly, it conflicts with no theological system, except when such a system forbids enquiry into Nature's methods, or would extinguish the torch of truth which it is the duty of each generation to hand on to the next.^13^

Readers were evidently anticipated to expect a conflict between eugenics and religion, in contrast to which the editorial stated that the journal would include areas of ‘religion, in so far as it strengthens and sanctifies the sense of Eugenic duty’. Religion was deemed significant enough to feature alongside only four other subjects that were planned for the new journal, ‘biology’, ‘anthropology’, ‘politics’ and ‘ethics’. Even areas that were seemingly unrelated to religion, such as the question of ‘heredity and environment’, were not free from reassurances to religious audiences, and the editorial stated the society's intention was to: ‘stimulate public opinion in favour of that wholesale desideratum, a sound mind in a sound body, without, however, neglecting the cultivation of the spiritual side of human nature, which is quite as important as its material side.’^14^

Following this introduction to the journal, Revd. W. R. Inge DD provided a much more detailed analysis of the ethical aspects of eugenics in an article titled ‘Some Moral Aspects of Eugenics’.^15^ This was an important religious endorsement for the society. Inge was raised in the high-church tradition, but in his adult life he embraced modernism and turned away from Tractarianism.^16^ In 1899, Inge delivered the Bampton Lectures, on the subject of ‘Christian Mysticism’, in which he discussed his belief that the church should not focus on claims about the miraculous but upon personal experience of God and prayer.^17^ In 1907, he was appointed as Lady Margaret Professor of Divinity at the University of Cambridge, and in 1911 he became dean of St Paul's Cathedral in London. Inge was a prolific author, and reached a huge audience through his weekly columns in the *Evening Standard*, which appeared between 1921 and 1946.^18^ Some of his contributions to periodicals, including work on eugenic ideas, were reprinted in collected volumes.^19^ Inge would later gain an international reputation as a Christian proponent of eugenics, but he made his support for eugenics clear from a very early stage in the history of the EES.^20^ Inge's contribution to the first issue of *The Eugenics Review* certainly endorsed the potential power of eugenics; he argued that the challenge was not the technical aspect of eugenics, but rather the difficulty of deciding which traits would be most socially advantageous. He provided an amusing illustration of this problem by questioning whether it would be best to breed ‘human mastiffs’ as police, or ‘human greyhounds’ as postmen.^21^

The positive gains that could be made through eugenics were only part of Inge's message, however, as he reminded readers that human interference with ‘natural selection’ posed a threat: ‘if nature is not allowed to take her own way of eliminating her failures, rational selection must take its place … Humanitarian legislation, or practice, requires to be supplemented, and its inevitable evil effects counteracted’.^22^ Inge made this statement with direct reference to his position as a member of the clergy, and noted that ethics had, for the ‘majority of people’, ‘a religious sanction, or even a religious foundation’.^23^ With this in mind, Inge described ways in which he believed eugenics fulfilled true Christian morality. Inge argued that people in ‘Class I’ had neglected ‘the chief duty which God and his country required of him’ when they chose not to have children. He attacked ‘any supposed interests of Christian morality’ which failed to challenge ‘degeneration’, and said the height of Christian ethics was found in Christ's command ‘“Be ye therefore perfect, even as your Father in heaven is perfect”’.^24^ With eugenics thus given the sanction of Christ, Inge looked to Christian history to develop his argument in a more disturbing manner:

Christian ethics does not (as is often supposed) teach the duty of preserving and multiplying life at all hazards. Once convinced that so-and-so was an undesirable citizen, the Church … lost no time in hurrying him out of the world. No doubt they usually burnt the wrong people, which was very unfortunate; and you must not suppose that I want to see *autos da fè* even of our most degraded specimens; but my point is that there is nothing inconsistent with Christianity in imposing as well as enduring personal sacrifice where the highest welfare of the community is at stake.^25^

It does not easily escape notice that Inge's argument here required him to clarify that he was not presenting a Christian justification for burning people alive; but next to this brutal image Inge stated that to ignore the ‘modern weapons’ of modern science, which offered new ways to tackle ‘evil’, would be ‘not Christian … only barbarous and mediaeval’.^26^ This spirited, and heavily moralised, appeal for eugenic intervention reflected Inge's theological views. Inge attacked the idea of a conflict between ‘science and religion’, stating that this was based in a ‘false philosophy’ of ‘materialism’, but he also argued against those who would uphold ‘picture-book theology’ in the face of ‘the facts of science’.^27^ For Inge, this included the issue of the miracles in the Gospels; he argued that this was ‘a scientific and not a religious question’.^28^ It is clear that Inge did not believe it was wise, or ethical, to create false divisions between science and faith. His argument that Christianity sanctioned ‘sacrifice’ was consistent with his claim, almost Darwinian in tone, that ‘struggle’ was necessary in order to obtain ‘Goodness’:

It is the paradox of the spiritual life that if we could take to ourselves ‘the wings of a dove’ and escape from this world of mingled good and evil, we should not reach the rest which we desire. For one at least of the Divine values, Goodness, cannot be realised by flight, but only by struggle.^29^

Up until this point the relationship between religious groups and the EES appears to have been fairly straightforward. Religious figures were approached in the hope that they would lend support to the eugenics movement, and the impact of Christian teaching upon eugenics was discussed with a view to reconciling one to the other. A parallel debate, and one which exerted significant influence upon EES policy, was the decision to ensure that eugenic strategies did not cause unnecessary offence to religious supporters. The third issue of *The Eugenics Review* featured an article by another prominent cleric, Revd. J. H. F. Peile, who had delivered the 1907 Bampton Lectures at the University of Oxford.^30^ Peile argued that there was nothing remarkable about clerical support for eugenics, and suggested there were only two reasons that the movement did not receive active support from more ministers. The first was purely practical; clergymen were busy. The second obstacle was more serious, eugenics had not been presented ‘without offence’ and ‘popular opinion’ associated it ‘with things which no Church can possibly touch’.^31^ Peile suggested that the EES should focus upon correcting misinformation surrounding eugenics, thus ensuring the ‘ground is prepared’ for clerical support, which he believed was merely an extension of ‘a principle to which the Church is already committed’ in therapeutic philanthropic work.^32^ The EES evidently agreed with this strategy, as after this message had been printed in *The Eugenics Review* it was later agreed to publish 1,000 extra copies of the text for circulation.^33^

There is some disparity between Peile's claim and the more recent historiographical assessment that eugenicists sought the forceful replacement of traditional ethics. Francis Galton, who was first to use the term ‘eugenics’, has often been used as evidence of this conflictual relationship, described as presenting ‘racial regeneration’ in ‘fashionably scientific terms’ rather than ‘traditionally moral ones’.^34^ Galton was certainly not an advocate of cautious or diplomatic relations with religious believers; in 1872 he published a statistical denial of the power of prayer, and he praised Charles Darwin's work for having dispelled Christianity like a ‘nightmare’.^35^ Galton was not alone in this respect, and Marius Turda has provided interesting examples of individuals in the Hungarian eugenics movement who called for a new morality based on biology as a replacement for older religious values.^36^ Turda has noted that secular theories of human improvement came into most conflict with religious groups as they pressed for legislative changes in areas such as marriage, which were traditionally governed by the Church.^37^ In many cases, this was described as a new ‘eugenic religion’, although this was often done in a vague rhetorical manner.^38^ Galton suggested that the term ‘religion’ could be applied ‘to any group of sentiments or persuasions that are strong enough to bind us to do that which we intellectually may acknowledge to be our duty, and the possession of some form of religion in this larger sense of the word is of the utmost importance to moral stability.’^39^ For Galton, eugenics needed to promote ‘the religious significance of the doctrine of evolution’.^40^ John Waller has linked this theme to the desire of Galton to create a ‘scientific clerisy’, or a professionalised group with social prestige to rival that of the church.^41^ Waller states that this ambition was directly related to Galton's involvement in the prayer-gauge debate; Galton promoted a naturalistic view of the world and resented the monopoly which the church had enjoyed in the field of education.^42^

Julian Huxley, grandson of Darwin's bulldog T. H. Huxley, argued that religion should not be abandoned but rather transformed in the light of scientific advance. He refused to reject the significance of religious feeling and viewed evolution as progressive, with man as its best outcome and still retaining a moral purpose.^43^ These views did not prevent Huxley from criticising existing forms of religious belief. His most famous text on the subject, *Religion Without Revelation*, first published in 1927, expressed his belief that ‘religion of the highest and fullest character’ could exist without belief in ‘a personal god’. He suggested that this was merely a change of ‘clothes’, but presented a stinging attack on ‘priests’ and ‘religious organisations’ ‘feather[ing] their nests out of the pretended supernatural power which they wield’; he dismissed the ‘hideous terror of everlasting hell’, which was said to force people ‘into the channel of propitiatory sacrifice’, and the ‘meaningless mumbo-jumbo of certain types of ritual’. Instead of these flawed expressions of faith, Huxley suggested a reformed religion to be based on ‘the three-fold basis of agnosticism, of evolutionary natural science, and of psychology’.^44^ However, this eugenic religion was not always viewed as a replacement for Christian faith. R. A. Fisher, famed as one of the founders of the neo-Darwinian synthesis and a deeply religious man, viewed eugenics as a natural addition to his Christian faith. James Moore has argued that Fisher viewed eugenics as a form of salvation, part of his Christian faith that required expression in works.^45^ Moore has described this belief as Fisher's ‘passionate credo’, a statement of faith that was voiced in 1912 at the Cambridge Branch of the EES:

We require a new pride of birth, in that whatever valuable quality we show really goes to establish the quality of the family; and a new confidence in our instinctive judgments of human worth. As the Dean of St. Paul's says, ‘We need a new tradition of nobility’.^46^

In the case of Inge, the Dean quoted at this meeting in Cambridge, and that of Fisher, ‘the religious significance of evolution’ was not regarded as in opposition to Christian belief. It was this understanding of faith, and not the aggressive secular vision of Galton, that appears to have been predominant within the EES. Montague Crackanthorpe, the first president of the EES, made religiously charged references to Galton as ‘Founder’ and ‘the chief Apostle of Eugenics’, but the society distanced itself from Galton's more provocative religious arguments.^47^ In 1909, when the EES received permission to ‘publish a collection of his [Galton's] papers in book form’, it was decided to include:

- Probability, The Foundation of Eugenics
- Eugenics, its Definition, Scope & Aims
- Restrictions in Marriage
  Studies in National Eugenics
  Eugenics as a Factor in Religion
- The Possible Improvement of the Human Breed etc.
- Local Associations for promoting Eugenics.^48^

When Galton had published these essays in 1883 there were three additional chapters, ‘Objective Efficacy of Prayer’, ‘Enthusiasm’, and ‘Possibilities of Theocratic Intervention’. All of these, however, were removed by the EES and only reappeared when the society republished the collection of essays in 1951.^49^ Galton's most provocative and incendiary essays, those cited as examples of eugenicists' desire to replace traditional religious beliefs with a secular morality, were avoided by the EES. ‘Eugenics as a Factor in Religion’ was much tamer, and emphasised that ‘eugenics strengthens the sense of social duty’ and ought to find ‘a welcome home in every tolerant religion’.^50^ A similar caution with respect to religion marked the interaction of the EES with the international eugenics movement. In October 1911, when the society met with Dr. Ploetz of the ‘German Society for Race Hygiene’, it was agreed to form an ‘International Race Hygiene Society’ to assist preparations for the ‘proposed International Congress of 1912’.^51^ One of the three principles considered essential to the success of this endeavour was the agreement that: ‘The Society hold itself aloof from party strife and party differences whether political or religious’.^52^

The issues identified by Turda as particularly troublesome for the relationship between religious communities and eugenics organisations, such as sex and marriage, were those about which the EES appears to have been especially cautious. The same meeting that set out sensitive plans for forming an International Race Hygiene Society agreed that venereal disease was not a subject that should ‘for the present’ appear in *The Eugenics Review*; however, the subject was not to be ignored, but ‘as much unobtrusive work as possible should be undertaken’.^53^ Some historians have suggested that the subject of venereal disease was a distraction for the EES, and there were times the society did question whether the subject was truly a eugenic issue, but at this stage the primary concern was that of public opinion.^54^ In November 1911, the EES unanimously agreed to ‘urge the consideration of the subject on the medical bodies’ but to refrain from ‘definite public action on behalf of the Society’.^55^ The same principle was repeated in February 1912 when it was agreed that ‘any recommendation of individual medical men to give definite advice in any way connected with the Society as to the fitness of marriage of individuals would be extremely dangerous’. It was, however, recognised that the growing sentiment in favour of such examinations should be ‘cordially encouraged by the Society’.^56^ The matter in question was not whether these issues were truly eugenic, but how treatment of these issues would impact upon the popularity of the EES. These concerns were central to the plans formed by the EES for reconstruction after the First World War. Issues including ‘housing’, ‘divorce’, ‘emigration’ and ‘maternity assistance’, were all considered as possible areas of eugenic action, but it was agreed that any support for legislative action needed to fulfil two criteria:

In the first place official action should only be taken in regard to measures of a directly eugenic character. And secondly that the Council should not attempt to take action as a whole in regard to those reforms which may have only an indirect eugenic bearing, but which must necessarily be the ground for strong differences of opinion between members of the society on religious, political, or moral grounds.^57^

The EES discussed ‘Divorce’ as an example of a contentious issue that had a secondary eugenic relevance. In this instance a cautious, entirely neutral, resolution was passed: ‘That in regard to the reforms connected with marriage laws, the aim of the society should be to increase the sense of parental responsibility, and thus to promote care in selection in marriage.’ Even with that muted statement, which totally bypassed any direct discussion of divorce, EES minutes reveal there was still one ‘dissentient’ and one abstention.^58^ External propaganda work appears to have been guided by similar considerations, and discussions for the 1917 ‘Baby Week’ agreed: ‘diagrams and posters should be prepared illustrating those Eugenic facts which the Committee agreed it was advisable to present to the public’.^59^ The question was less what constituted eugenics, but which of those issues was advisable to present to the public. There was certainly evidence that this type of discretion was warranted. The EES experienced co-ordinated opposition from Catholics and the Labour Party when it tried to persuade the British government to legalise voluntary sterilisation in the interwar period.^60^ It was in this period that one of the most prominent Catholic opponents of eugenics, G. K. Chesterton, with a readership to rival that of Inge, launched a scathing attack on eugenics:

The Eugenist doctors … do not know what they want, except that they want your soul and your body in order to find out. They are quite seriously, as they themselves might say, the first religion to be experimental instead of doctrinal. All other established Churches have been based on somebody having found the truth. This is the first Church that was ever based on not having found it.^61^

It is clear that the message about a religion of eugenics had been received, but not always in a positive light. When eugenicists were clearer as to their intentions, such as when seeking sterilisation legislation, there was an even stronger opposition from Catholics.^62^

Recognition of this feature of EES deliberations sheds light upon aspects of the debate surrounding the emergence of ‘reform’ over ‘mainline’ eugenics. The notion of ‘mainline eugenics’ as distinct from ‘reform eugenics’, the latter of which was said to have moved away from espousal of race and class bias, was first forwarded by Daniel Kevles and Richard Soloway.^63^ Pauline Mazumdar has provided the most compelling analysis of this issue in British eugenics. Her account details the call that was made inside the EES for a move away from genealogical depictions of basic eugenic principles, towards advanced statistical genetic analyses designed to uncover the relative influences of environment and heredity. However, in spite of this internal criticism within the EES, Mazumdar is cautious to note this was not a fundamental rejection of the principles of eugenics; furthermore the calls for reform were met with limited success as ‘mainline’ eugenicists continued to exert influence into the 1930s.^64^ It is also important to recognise that the EES consistently expressed concern over policies and actions that might attract external criticism. Recent research has suggested that one of the features of the ‘reform’ period was a greater willingness in the EES to form ‘coalitions [with external groups] … for strategic purposes’.^65^ It is certainly true that the EES made concerted efforts to co-operate with external organisations during this later period, but there had been frequent interactions with external groups throughout the history of the EES.^66^ The care that the EES took to avoid unnecessarily offending religious believers reflected a more general attempt to create a ‘broad church’ of support for eugenics. An examination of EES minutes reveals that the society met with 59 separate external organisations in the period between 1907 and 1935; of these there were 34 meetings between 1908 and 1920, and 26 between 1921 and 1935.^67^ The distinction between these two periods was much less pronounced for the EES than some accounts might suggest.

The pursuit of harmonious relations with individuals and organisations external to the EES was also a policy that caused internal tensions across the period covered by the ‘mainline’ and ‘reform’ periods of eugenics. In 1915 ‘Dr Drysdale’, a prominent advocate for birth control measures, resigned her membership of the EES in protest at its policy on ‘Malthusian propaganda’.^68^ Her complaint was that the EES was too timid in its support for birth control, but the society responded by stating that ‘there were many different ways in which the Eugenic problem could be attacked, and that it was possible for various groups of labourers to continue to work, not inharmoniously, in different fields.^69^ The policy of discretion, later made explicit in EES discussions about post-war reconstruction, appears to have guided this internal division; it was acknowledged that eugenic good might be done in work carried out by societies external to the EES, but some issues were considered too high risk to be given public support. Drysdale's criticism was echoed in 1932 by George Pitt Rivers, an EES member who was known for his far right political views and anti-Semitic political and anthropological writings.^70^ Pitt Rivers argued that the society's caution had backfired, and suggested that even strong criticism of the organisation could serve a useful function:

it does not follow that the interest aroused by controversy does not strengthen the side with a strong and logically presented case immeasurably more than compromising and equivocal attempts to conciliate all views or timorous avoidance of any subject or point likely to evoke opposition, which is invariably rewarded with indifference or contempt.^71^

The provocation for this outburst was the rejection of his proposal to have a public ‘debate between two or more speakers on “Religion and Eugenics”’, which the EES deemed more suitable as an ‘informal and private gathering’. Pitt Rivers argued that this type of debate was important because the interactions of eugenics with ‘politics’ and ‘religion’ were ‘two of the principal sources of interest in, as well as some of the organized opposition’ to the eugenics cause. His anticipation of ‘controversy’ was not hypothetical, but he argued that the EES policy of conciliation and caution had ‘lost far more than it had gained in influential support’. A final stinging criticism argued that controversy, ‘restrained only by a regard for scientific conventions and friendly feeling’, is a symbol of ‘the most healthy, vigorous and influential scientific societies’. By avoiding controversy for the sake of popularity, Pitt Rivers insinuated that the EES was undermining its scientific credentials. Certainly there was truth that the EES had consistently limited its engagement with controversial issues out of a desire to maintain, or create, popular and widespread support for eugenics. Religious groups were a large part of this concern, and in March 1930 the EES Committee passed a motion to remind the editor of *The Eugenics Review* about the need to consider the opinions of ‘medical men and clergymen’ when publishing book reviews. In particular it was ‘recommended’ the editor ‘should bear in mind the existence of a large body of readers whose moral opinion it is important not to offend’.^72^ By being placed next to medical authorities, clergy were already given a position of great prominence and authority, but this was further emphasised as the final resolution only considered ‘moral opinion’. In this instance, and many of the preceding examples, religious organisations were not only groups with whom it was desirable to enjoy cordial relations, but rather the concerns of religion dictated the activities and ideas that the EES were willing to embrace. In some sense, therefore, it is possible to suggest that religious communities helped to define what eugenics came to mean—at least in the public propaganda work of the would-be official spokespeople of eugenics.

## The American Eugenics Society

The experience of the EES was far from unique, and an analysis of the papers of the American Eugenics Society (AES) demonstrates that religion was a consistent preoccupation for the eugenics movement in the United States. Christine Rosen has provided a useful account of the way in which the AES sought to employ religious imagery and publicise its work on the back of clerical support.^73^ However, in this section it will be argued that concerns about the supposed ‘misinterpretation’ of eugenics within religious communities prompted the AES to employ what may be termed ‘negative definitions’ of its activities. Ultimately AES refutations of activities with which it did not wish to be associated came to occupy more space than positive statements of true policy. In looking at this issue, I offer an addition to the historiographical debate surrounding the concept of ‘reform eugenics’ versus the idea of the continuity of negative and racist elements within American eugenics. It will be argued that concerns over the alleged misrepresentation of eugenics, and the associated fear of religious criticism, resulted in a conscious move towards a more softly spoken form of eugenics. Rather than replacing previous ideological beliefs, however, it will be argued this was merely intended as a temporary measure designed to allow popular opinion to be won over and the new eugenic faith to erase former doubts.

As in the EES, religion was recognised as an issue that required sensitive handling from the very beginning. The AES grew out of and replaced the Eugenics Committee of the United States of America, which in turn had been formed in response to the Second International Congress of Eugenics held in New York City in 1921.^74^ Henry Fairfield Osborn, who had secured the American Museum of Natural History as the venue of the 1921 Congress, wrote to Leonard Darwin of the EES less than three months after the Congress to state:

I have the best possible news for you, namely, the hearty endorsement of the Eugenics Congress by the leading Roman Catholic prelate in America, Archbishop Hayes of the Diocese of New York. … On every side there is evidence that the eugenics propaganda has taken a firm root in this country. For the first time people understand what we are driving at and sympathize with the movement.^75^

Perhaps partly to build upon this sense of good will, but again reflective of a similar idea discussed by the EES, Osborn recommended a motto for the AES that would emphasise its religious qualities: ‘To raise American civilization through improving the spiritual, intellectual, moral and physical characters of the American people’.^76^ Osborn was a very high profile figure in this period, to the extent that Brian Regal suggests his reputation as a scientist was second only to that of Einstein.^77^ His interest in emphasising the religious or spiritual value of eugenics was mirrored by his beliefs about evolution more generally. Regal demonstrates that Osborn was committed to a form of ‘soft-view predestination’ regarding evolution; he believed that an organism's characteristics were guided by its constitution, but could be liberated from this plan through struggle. This idea became fused with Osborn's religious views as he equated the spiritual struggle for salvation with the physical struggle for evolutionary advancement.^78^

Osborn shared these ideas with James McCosh, a friend of his from Princeton University, who subscribed to a form of evolutionary Calvinism. David Livingstone has argued that McCosh played a critical role in preparing the ground for the theological acceptance of Darwinian theory. McCosh promoted the idea that nature was guided by laws, and that all parts of nature were interconnected, with an immanent God.^79^ Crucially, for the Scopes Trial, to be discussed later in this paper, McCosh was a pioneer in the teaching of evolution in America, but he fused this with a teleological understanding of the world.^80^ Regal has argued that these religious views are crucial if we are to understand Osborn's scientific beliefs, and this certainly appears to be justified by an examination of his interaction with the eugenics community. Osborn believed that the religious motto was so important that when he could not attend meetings of the AES, due to ‘arrears’ in his ‘regular duties’, he reiterated the idea in a letter to the committee. He was very keen that the motto should not be held up as a consequence of his absence.^81^

The Committee did not implement a motto, but when members were sought for an ‘advisory council’ a special effort was made to incorporate members of the clergy. These names were printed on AES publicity, such as the letterheads where Osborn had hoped to place the religious motto, and the ‘advisory council’ was given power of veto concerning AES propaganda work.^82^ Although a letterhead from 1923 included only five clergymen out of a total of 99 names, other promotional material gave clergy a much more prominent position.^83^ Kenneth Ludmerer's analysis of the AES booklet *What I think About Eugenics* has revealed that of the 143 people quoted in favour of eugenics, only eight were trained in genetics while there were 19 members of the clergy.^84^ The high proportion of clergy involved in this publication reflects an organised strategy designed to ensure their support. In February 1923, the AES developed its outreach to groups of particular eugenic significance by organising committees dedicated to ‘co-operation’ with ‘Physicians’, ‘Social Workers’ and ‘Clergymen’. Alongside these groups there were committees organised around matters of strategy and policy, for example ‘selective immigration’, ‘research’, ‘exhibits’, ‘biologic geneology [sic]’, ‘crime prevention and legislation’, ‘finance’ and ‘organization’. The Committee on Co-operation with Clergymen entertained ambitious plans of creating a committee of 40 members overseen by a ‘small executive committee’, and possessed a large share of the AES budget accordingly.^85^ However, even committees not devoted to religion were faced with evidence of the importance of securing positive relations with Christian believers. A survey conducted by the AES ‘Committee on Formal Education’ received 162 suggestions as to which students ‘the subjects of genetics and eugenics might properly be required’. The most commonly suggested group was ‘theological students’ with 61 votes, followed by ‘teachers’ with 34, and students of ‘psychology’ and ‘agriculture’ who received 12 votes each.^86^

Religious ideology was also incorporated into one of the most successful outreach strategies employed by the AES, that of the ‘fitter family contests’ that were held at fairs across the nation. Robert Rydell has argued this scheme tapped into the enormous popularity of world's fairs, and suggested that eugenicists became ‘showmen’ in this context.^87^ Certainly the records that survive from these events suggest that several thousand people voluntarily entered themselves into contests to be assessed on their eugenic worth, based on issues as diverse as their ‘thriftiness’ to ‘organic … functional disorders’.^88^ In 1928, there were requests for 42 fitter family contests; with the papers that survive from two contests in 1927 each recording over 100 entrants examined, it would not be unreasonable to estimate over 4,000 entrants in 1928 alone—and this figure does not include the large number who would have witnessed these ‘shows’ as spectators.^89^ It is significant, therefore, that the AES chose to bestow the accolade of eugenic fitness to the winners of these contests with a biblical motto that Madison Grant had been appointed to find: ‘Yea, I have a goodly heritage’.^90^

With this important position ascribed to religious communities and ideas, criticism from this sector of society was taken especially seriously. This concern was reflected in one of the most popular and enduring works undertaken by the Committee on Co-operation with Clergymen, ‘a booklet containing 100 questions and answers’ designed to ‘clarify the minds of clergymen, too many of whom think that eugenics is birth control and nothing else’.^91^ This booklet, later named *A Eugenics Catechism*, was a regular feature of AES policy discussions, and evidence suggests thousands of copies were distributed across the United States.^92^ The AES member Leon Witney claimed ‘thousands’ of these booklets were ‘circulated … to colleges and to organizations—churches for example—who asked for them’.^93^ Seventeen folders of correspondence survive from individuals and organisations that requested copies of the ‘catechism’, a total of 343 letters. Of these surviving letters requests came from 42 different States, as shown in Table 1, suggesting an influence diffused across the nation.

**Table 1.** Letters received by the AES requesting *A Catechism of Eugenics*, listed by State

New York	57	Oregon	7	Maine	3	South Carolina	1
California	36	Missouri	7	Connecticut	3	Alabama	1
Ohio	29	Texas	6	New Hampshire	3	Utah	1
Pennsylvania	23	Colorado	5	Nebraska	3	Nevada	1
Illinois	18	Wisconsin	5	Maryland	3	Idaho	1
Iowa	17	Florida	4	North Carolina	3	Virginia	1
New Jersey	16	Washington	4	Arizona	2	West Virginia	1
Michigan	14	Hawaii	4	North Dakota	2	Montana	1
Massachusetts	13	Vermont	4	Georgia	2	Oklahoma	1
Kansas	10	Minnesota	4	Rhode Island	1		
Indiana	7	Louisiana	3	South Dakota	1		

*Note*: There was also one request from Washington DC, and further afield 12 from Canada, one from the UK, and one which could not be identified. AESP Box 1 and Box 2.

The context of the *Catechism*'s creation was markedly different from that of Henry Fairfield Osborn's former jubilation at clerical support. In 1925, the year the *Catechism* was first discussed, Osborn was involved as an expert for the defence at the ‘Scopes Trial’.^94^ The Scopes Trial was, on the surface, a debate about the teaching of evolution in schools, but this subject was bound up with the issue of eugenics, as well as the competing forces of fundamentalism and anticlericalism. As the teaching of evolution filtered into schools, there was a need for textbooks to cover this subject; the textbook implicated in the Scopes Trial was George William Hunter's *A Civic Biology*.^95^ This text included a discussion of eugenics, which Hunter described as ‘the science of improving the human race by better heredity’. A section that discussed those considered to be eugenically unfit stated that ‘If such people were lower animals, we would probably kill them off to prevent them from spreading’. Hunter recognised that this would not be accepted in the case of man, but suggested that forms of segregation should be enacted to prevent ‘perpetuating such a low and degrading race’.^96^ William Jennings Bryan identified the eugenic ideology as ‘brutal’, and this has been seen as one of the key issues leading him to turn against evolutionary theory.^97^ Although Bryan was not strictly a biblical literalist, Osborn got into a heated debate with him and stood firmly opposed to the rise of fundamentalism.^98^

The concern that fundamentalism might obstruct the path of scientific education was combined in the *Catechism* with a fear that eugenics was being misidentified and misappropriated by other groups, with a detrimental impact upon its popularity. The first question answered in the *Catechism* was, rather sensibly, ‘What is eugenics?’ A definition from Francis Galton was quoted in reply: ‘Eugenics is the study of those agencies under social control which may improve or impair the inborn qualities of future generations of man either physically or mentally.’ Perhaps more surprisingly this was immediately followed by 15 questions as to whether eugenics involved various other issues, all of which were denied. Ten of these received a simple ‘no’, the AES did not consider eugenics to be related to ‘sex hygiene’, ‘prenatal culture’, ‘public health’, ‘free love’, ‘trial marriage’, ‘a vice campaign’, ‘government-made marriage’, ‘physical culture’, ‘Spartan infanticide’ or ‘breeding human beings like animals’. The remaining five questions, surrounding ‘birth-control’, ‘producing genius to order’, ‘making supermen’, ‘scientific love making’ and ‘less love in marriage’, required more nuanced replies but the general answer was the same, ‘no’, or not in the way they believed most people would think. Of birth control it was said:

Q. Is eugenics birth control?

A. No, not in the sense in which the term is commonly used. The conception of fewer inferiors is eugenic, but such birth control as reduces the conception of superiors is opposed to eugenics.^99^

This rather equivocal answer was developed later in the *Catechism* where, in contrast to the above position, it was explained: ‘The control of births is the principal means of improving the stock.’ Once various means of birth control had been explained, the matter of religious opinion was raised. The answer was by no means as positive as the AES might have liked:

The Protestant and Jewish Churches have taken no definite position. The Roman Catholic Church tolerates birth control accomplished by marital continence, or the use of the “safe period,” but opposes the use of contraceptives.

As if to combat this mediocre support from religious organisations, the questions that followed were directed at setting forth the high purpose of eugenics:

Q. What is the most precious thing in the world?

A. The human germ plasm.

Q. How may one's germ plasm become immortal?

A. Only by perpetuation through children.^100^

In the midst of controversial ethical issues, which divided opinion, the AES determined to portray the eugenics movement as one with undeniably noble aims. The final four questions in the definition section of the Catechism addressed the relationship of the Christian faith towards this noble eugenic cause. The first, ‘Does eugenics contradict the Bible?’, might have been expected to elicit a straightforward ‘yes’ or ‘no’ given the previous fifteen questions, but the reply was: ‘The Bible has much to say for eugenics. It tells us that men do not gather grapes from thorns and figs from thistles.’^101^ For those left in any doubt, the next answer addressed the question of whether eugenics was antagonistic to the Bible. Unsurprisingly, the answer given was ‘no’; instead it was argued ‘The aim of eugenics is to insure the totality of human welfare in the long run.’ The final two questions in this section were not specifically addressed to Christian readers, but the subject left little room for doubt. Connected to the previous subject of ‘human welfare’, the third question discussed the virtue of charity and whether eugenics meant ‘less sympathy for the unfortunate’. This subject received one of the most detailed answers of the entire Catechism.^102^ Rather than being an unkind or cruel system, it was said eugenics would lead to a ‘better understanding of them [the unfortunate]’ and more effort ‘to alleviate their suffering’. The method of this kindness was self-confessedly different, however:

by seeing to it that everything possible is done to have fewer hereditary defectives … fewer unavoidable unfortunates with which to divide a sympathy which should be more fully and effectively expended on the inevitable unfortunates. … This is a true kindness both to the victims and to society.

In the final instalment of this ‘negative definition’ of question and answers, the tense subject of evolution was addressed:

Q. Must one who believes in eugenics believe in evolution?

A. Yes, that evolution is a present and a continuing process. It is not necessary to believe that the original or ancestral man evolved from apes. All admit that there has been an evolution in the differentiation of the races, and from fossil man to modern man. Should we not want more of such evolution?^103^

Whilst this answer is uncompromising in its ‘yes’, the first positive reply of the Catechism, it seems that the force had been dampened in line with Henry Fairfield Osborn's views rejecting simian ancestry. His campaign over this issue was strongest in 1926, the year this version of the *Catechism* was published, and there was an added ethical dimension as Gowan Dawson has noted the concept of simian ancestry was connected with prominent attacks upon evolution based upon fears of sexual morality.^104^ In rejecting a controversial aspect of evolutionary theory, and one associated with sexual propriety and religion, the *Catechism* emphasised it was not necessary to believe in unpleasant origins of mankind—only those facts which allowed for his future advancement.

The presentation of eugenics as a movement with noble qualities comparable to religious ideals was not unique to the AES. *Eugenical News*, identified as a ‘mainline eugenics’ periodical, was certainly supportive of this religious presentation of eugenics.^105^ Dr W. A. Dorland, professor of Gynaecology in Chicago, stated that eugenics was ‘beyond reproach’ but suffered because it remained ‘visionary’. Dorland argued that the only people who would willingly forgo marriage on the grounds of their eugenic inadequacy would be those ‘in whom the moral and religious senses are predominant, the stuff of which martyrs are made’. The scarcity of such people led Dorland to call for the sterilisation of all ‘degenerates’.^106^ In 1918, an article stated that it was a State ‘sin’ for parenthood not to be made an economic asset for families; and in 1923, it was argued that the timescale necessary for the benefits of eugenics to be seen meant that the discipline should be considered ‘like the founding and development of Christianity, something to be handed on from age to age’.^107^

However, these types of comparisons were not always intended as praise. The eugenicist Charles Davenport argued that the greatest threat posed to eugenics was ‘from some impetuous temperament, who, planting a banner of eugenics, rallies a volunteer army of utopians, free lovers, and muddy thinkers to start a holy war for the new religion’.^108^ Davenport's position as a research scientist no doubt influenced this attack upon a ‘volunteer army’, but the charge was that there were some for whom the message of eugenics was more extended than the evidence used to substantiate it.^109^ The AES was certainly aware of the danger posed by this charge, and in 1927 a draft report issued by the ‘Committee on Formal Education’ met with criticism for its claim that ‘a teacher of eugenics should not be an enthusiast’. The meaning, that teachers should not abandon ‘objectivity and soundness’, was accepted, but the committee felt that this concern should not be raised outside the society.^110^ Criticism continued, however, and fears over the quality of eugenic research featured heavily within AES policy under the influence of Frederick Osborn, the nephew of Henry Fairfield Osborn. In 1933, Frederick Osborn wrote a confidential ‘Memorandum on the Eugenics Situation in the United States’, which complained that the AES had ‘started on a sort of Galtonian propaganda without having enlarged their base of factual and experimental studies’.^111^ This criticism is significant as Frederick Osborn has been identified as a key proponent of ‘reform’ eugenics in the United States.^112^ Barry Mehler has criticised this thesis, and objected to the sharp division of eugenics into periods of ‘old’ versus ‘new’, noting that American eugenicists continued to be influenced by racial issues in the 1930s.^113^ Recent scholarship has added to this criticism and emphasised a continuity of activities and ideology in the movement during the 1930s;^114^ however, rather than being forced to choose between competing interpretations, the theses can be reconciled by recognising that the movement did change in this period but the changes were essentially cosmetic in nature.

One of the schemes inaugurated by the AES following the appointment of Frederick Osborn to the Board of Directors, in May 1935, was a series of conferences in association with ‘persons engaged in efforts for social betterment along lines whose connection with eugenics has not been sufficiently realized’.^115^ A recurring theme at these conferences was the policy of advancing a discreet and mild-mannered form of eugenics. A. E. Wiggam opened the ‘Conference of Publicists’ in December 1937 by noting that those who had been involved with eugenics for a number of years were aware of being subjected to ‘the most critical examination’. He went on to speak of the need for an ‘evangelistic’ element to eugenics, to ensure the ideology became ‘not only our common objective, but also the spiritual ideal and the structural and dynamic object of Society itself’. What is remarkable in this proposal (which spoke of eugenics inspired by ‘tolerance that cuts across all class and racial lines’) was his conclusion that ‘the most effective way to write about eugenics is not to write about eugenics at all’.^116^ For those publicists who had made the effort to attend the conference, this might have seemed a disappointing conclusion. However, Wiggam did not call for the cessation of eugenic propaganda, but rather cited the example of a recent article in *Harpers* which, despite not using the word anywhere in its text, he argued made an ‘admirable plea for eugenics’.^117^ The eugenic cause was to be advanced by stealth and without attracting acrimony.

The ‘Conference on Education and Eugenics’ raised the same issue. The theme was raised in notes that were circulated to participants before the meeting:

One of the most serious problems that now confronts us is the fact that the word ‘eugenics’ has been widely misunderstood. … One of our first problems is to overcome these misapprehensions. Should this be done indirectly by teaching the fundamentals of eugenics without using the name? Or shall the term ‘eugenics’ fight its way into popular favor by repeated statements of what it really means?^118^

Essentially what was under discussion was whether the term ‘eugenics’ should be abandoned by a eugenics society. This remarkable policy has not received attention in any previous studies, and it is important to note that the context of opposition and misunderstanding under consideration was a religious one: ‘closely connected’ to the problem of:

how we can convince our Roman Catholic friends that while there may legitimately be diversity of opinion as to the use of certain methods, there can be no reasonable disagreement as to the basic tenet that the trend of reproduction ought to be altered.^119^

Former joy at Catholic support for eugenics had been replaced by disappointment as all these members resigned from the AES in response to the 1930 papal encyclical *Casti connubii*.^120^ In this context, the participants at the conference on education concluded in favour of discretion. Professor Henry Noble MacCracken of Vassar College, who described a popular eugenics course marketed to students simply as ‘marriage and the family’, pressed for subtle eugenic propaganda with a reference to biblical language. He argued ‘If the eugenist is to save his soul, he must first lose it.’^121^ Surreptitiously advancing eugenics thereby lost any taint of deception and instead took on a noble image of self-sacrifice and assumed religious authority in a parallel of Christ's words ‘whoever wants to save his life will lose it, but whoever loses his life for me will find it.’^122^

Frederick Osborn was also an advocate of this method of eugenic propaganda. He argued that the least controversial subject for eugenicists to promote was ‘parenthood’, and in 1936 he claimed: ‘By choosing the quality of the home in which children are reared as the measure for eugenic selection, all racial, social, religious and regional controversy is eliminated. No race and no social class has a monopoly on the quality of the home environment.’^123^ This was not a capitulation to the critics who emphasised the supremacy of environmental conditions, however, but a decision grounded in tactics. Addressing the AES in May 1938, Frederick Osborn acknowledged how difficult it was for ‘those of us who believe in the importance of eugenics’ to understand why the movement had not made more progress. He complained that ‘other than a very limited use of sterilization in some states’ little had been achieved.^124^ The ‘old’ eugenics was criticised not for its methods, but for its failure to implement them adequately. Frederick Osborn went on to argue that the movement had been hampered by its seeming focus upon a ‘multiplicity of purposes’; for this reason he suggested directing the organisation's energy to a single aim, that of raising the rate of births amongst ‘competent above-the-average people throughout the entire population of the United States’. He believed this would enable eugenics to become ‘a great popular noncontroversial and effective movement, with wide public support’.^125^ The emphasis here was upon popularity, but the old methods were not rejected; Osborn suggested that the promotion of controversial aspects of eugenics, such as sterilisation, should be outsourced to the medical profession.^126^ This satisfied Osborn's belief that the public would accept such intervention from doctors in a way they would resist from eugenicists, and his belief (expressed as early as 1934) that eugenicists should avoid all controversy until they had ‘won the support of the scientific workers and critical, public-spirited thinkers’.^127^ Controversial aspects of eugenics were not abandoned, they were simply to be conducted outside of the AES or postponed until public opinion was more receptive.

It was not only measures of negative eugenics that retained support from the AES. Although the society chose to speak less about race, the notion that racial ideas ceased to have support seems to have developed in the 1960s. In 1966, Frederick Osborn replied to a letter which complained about the ‘Government forcing school integration’ by stating that the thesis of ‘anglo-saxon’ superiority had been ‘thoroughly disproved by the scientific work of the last thirty years’.^128^ However, only twelve years prior to this, Frederick Osborn had written a ‘Eugenics Credo’ in which he criticised social scientists for failing to ‘take into account the factor of genetic variation’ and for helping to: ‘maintain popular beliefs that “bad” environments are the cause of all human ills, that all men are “equal”, that all races are “the same”.’^129^ Though this ‘Credo’ did not hold out the superiority of any one race, it was confident that mixture of ‘races’ was generally harmful; it was certainly not the ‘race’ free science that Osborn retrospectively claimed.

## Conclusion

It is clear that both the EES and the AES saw the issue of religion as a source of considerable concern. Each society made repeated attempts to appeal to religious communities for support, and great efforts were made in an attempt to ensure their work was not publicly offensive. The success of this public image management is highly questionable; the societies themselves frequently blamed external groups for tainting the name of their cause. This appears to have been the case even where they privately agreed with the controversial ideas that triggered this opposition. Aside from fear of the moral authority of religious groups, which it was recognised could help or hinder the eugenic mission, both societies recognised that the issue of Christian philanthropy was an area of natural intersection with eugenics. Both were keen to dismiss ideas of a callous eugenics that intended to end all kindness, charity, and love; rather it was claimed their work would end a great deal of needless suffering. In this sense eugenics was presented as a partner to traditional religious values even where it proposed radical new methodologies.

It is also clear that reasons beyond scientific advance or ethical reinterpretations alone helped to shape the periods defined as representing ‘mainline’ and ‘reform’ eugenics. There was a considerable element of tactical thinking at work, with efforts to shroud controversial eugenic methodologies from public view—at the very least until the movement became more established. Religious communities held a prominent position in these considerations. In conclusion, a cautionary illustration from the case of Madison Grant serves as a reminder of the need to look beyond actions to the motives guiding behaviour. In 1930, Grant, one of the most notorious racists of the American eugenics movement,^130^ wrote to Henry Fairfield Osborn to reveal his intention to write a new book ‘somewhat along the lines of The Passing of the Great Race’ on the subject of ‘The Nordic in America’. Grant reassured Osborn, however, that ‘the propaganda element in the other book served its purpose in contributing to immigration restriction, so this next one can be more moderate in tone’.^131^ Here racism was moderated not for reason of ethics, but because it had accomplished its aim.

## Funding

This work was supported by The Wellcome Trust [grant number: 083319]. Funding to pay the Open Access publication charges for this article was provided by the Wellcome Trust via its award to the University of Oxford.

## Footnotes

1 Edward J. Larson, ‘“In the Finest Most Womanly Way”: Women in the Southern Eugenics Movement’, *The American Journal of Legal History*, 1995, XXXIX, 137.

2 Daniel J. Kevles, *In the Name of Eugenics: Genetics and the Uses of Human Heredity* (Cambridge, MA: Harvard University Press, 1995), 5, 11, 21–22, and 49–51.

3 Christine Rosen, *Preaching Eugenics: Religious Leaders and the American Eugenics Movement* (Oxford: Oxford University Press, 2004).

4 See in particular the contradictory conclusions found in: Lyndsay Andrew Farrall, *The Origins and Growth of the English Eugenics Movement, 1865–1925* (New York: Garland Publishing, 1985), 53, and G. R. Searle, *Eugenics and Politics in Britain, 1900–1914* (Leyden: Noordhoff International Publishing, 1976), 13.

5 Mottier gives examples of eugenic practice enshrined in legislation, including sexual sterilisation procedures, the regulation of marriage through licenses, and child removal policies. Mottier notes that where local authorities were financially responsible for the provision of welfare, eugenic arguments grounded in economics had a greater appeal. Véronique Mottier, ‘Eugenics and the State: Policy-Making in Comparative Perspective’, in Alison Bashford and Philippa Levine, eds, *The Oxford Handbook of the History of Eugenics* (Oxford: Oxford University Press, 2010), 142–3.

6 Marouf Arif Hasian, Jr., *The Rhetoric of Eugenics in Anglo-American Thought* (Athens: The University of Georgia Press, 1996), 93–4. In the case of Germany, Sheila Faith Weiss has provided a detailed account of Catholic and Protestant involvement in eugenic practices. Weiss, *The Nazi Symbiosis: Human Genetics and Politics in the Third Reich* (Chicago: The University of Chicago Press, 2010), 60–5, 100,

7 See Rosen, *Preaching Eugenics*, 184–5.

8 Jonathan Rose, *The Intellectual Life of the British Working Classes* (New Haven, CT: Yale University Press, 2001), 215–16. Campbell criticised the traditional exposition of the fall and blood-atonement, and involved himself in the Social Gospel movement. The congregation at his church was large and his sermons were published internationally. Keith Robbins, ‘Campbell, Reginald John (1867–1956)’, *Oxford Dictionary of National Biography* (Oxford University Press, online edition, 2004).

9 Kenneth Ludmerer, *Genetics and American Society: A Historical Appraisal* (Baltimore, MD: The Johns Hopkins University Press, 1972), 2 and 17; and Paul Crook, *Darwin's Coat-Tails: Essays on Social Darwinism* (New York: Peter Lang, 2007), ix and 1.

10 *The Eugenics Review*, 1909, I (Apr.), inside front cover.

11 EES minutes, 12 February 1908 and 22 February 1908, SA/Eug/L.1, Wellcome Library, London. All records subsequently listed ‘SA/Eug’ are held at the Wellcome Library, London.

12 EES Minutes, 7 October 1908, SA/Eug/L.1.

13 *The Eugenics Review*, 1909, I (Apr.), 3.

14 *Ibid*., 3.

15 *Ibid*., 26–36.

16 Tractarianism, also known as the Oxford Movement, developed in the 1830s and pressed for a return to the doctrine of apostolic succession and a focus upon the sacraments as a source of divine grace within the Anglican Church. Inge objected to what he perceived to be the ‘fierce bigotry’ of the movement and endorsed more liberal theological interpretations. His politics and theology converged in the area of eugenics: Inge did not believe in state provision of welfare, which he feared would help the weak and undeserving, and opposed calls to apply Christianity to the social sphere. Inge believed that Christianity offered guidance for individual, personal life, not a model for social ethics. Matthew Grimley, ‘Inge, William Ralph (1860–1954)’, *Oxford Dictionary of National Biography* (Oxford University Press, online edition, Jan. 2008). On Tractarianism, see: S. A. Skinner, *Tractarians and the ‘Condition of England’: The Social and Political Thought of the Oxford Movement* (Oxford: Oxford University Press, 2004).

17 These lectures, held at St Mary's Church in the University of Oxford since 1780, were established in order to ‘confirm and establish the Christian Faith, and to confute all heretics and schismatics’. University of Oxford, Statutes and Regulations, <http://www.admin.ox.ac.uk/statutes/354-051a.shtml#_Toc28142805>, accessed 23 March 2013. Some of these lectures gained a very wide audience due to the controversial nature of the themes which they addressed. In 1884, the Broad Churchman Frederick Temple, who had contributed to the hugely controversial *Essays and Reviews* of 1860, delivered the Bampton Lectures on ‘The Relations between Science and Religion’. Temple, who later became Archbishop of Canterbury, argued that Darwin's work did not alter the ‘substance of Paley's argument’ for the existence of God. James R. Moore, *The Post-Darwinian Controversies: A Study of the Protestant Struggle to come to terms with Darwin in Great Britain and America 1870–1900* (Cambridge: Cambridge University Press, 1979), 220. H. M. Spooner, ‘Temple, Frederick (1821–1902)’, rev. Mark D. Chapman, *Oxford Dictionary of National Biography* (Oxford University Press, online edition, January 2008).

18 Grimley, ‘Inge, William Ralph (1860–1954)’.

19 For example, ‘The Birth-Rate’ and ‘The Future of the English Race’ in William Ralph Inge, *Outspoken Essays* (Fifth Impression, London: Longmans, Green, and Co., 1920).

20 Inge was invited to speak at the Second International Congress of Eugenics, held in New York City in 1921, and spoke at four ‘leading churches’ in the city during this visit. Rosen, *Preaching Eugenics*, 133.

21 *Ibid*., 28.

22 *Ibid*., 29.

23 *Ibid*., 30.

24 *Ibid*., 31 and 33.

25 *Ibid*., 34.

26 *Ibid*., 36.

27 W. R. Inge, ‘Confessio Fidei’, in Inge, *Outspoken Essays* (1922, Republished, Kessinger Publishing Company, 2003), 36–8.

28 *Ibid*., 50.

29 *Ibid*., 32.

30 James Hamilton Francis Peile, *The Reproach of the Gospel: An Enquiry Into the Apparent Failure of Christianity As a General Rule of Life and Conduct* (London: Longmans and co., 1907).

31 *The Eugenics Review*, 1909, I (Oct.), 164–5.

32 *Ibid*., 166.

33 EES Minutes, 15 October 1913, SA/Eug/L.3.

34 Diane B. Paul, *Controlling Human Heredity: 1865 to the Present* (Amherst: Humanity Books, 1998), 4, 10, and 31.

35 D. W. Forrest, *Francis Galton: The Life and Work of a Victorian Genius* (New York: Taplinger Publishing Co., 1974), 111, and Paul, *Controlling Human Heredity*, 32. The wider context of the debate on the efficacy and purpose of prayer is explored in Robert Bruce Mullin, ‘Science, Miracles, and the Prayer-Gauge Debate’, in David C. Lindberg and Ronald L. Numbers, eds, *When Science & Christianity Meet* (Chicago: The University of Chicago Press, 2003).

36 Marius Turda, ‘“A New Religion?” Eugenics and Racial Scientism in Pre-First World War Hungary’, *Totalitarian Movements and Political Religions*, 2006, 7, 303–25.

37 Marius Turda, *Modernism and Eugenics* (Houndmills: Palgrave Macmillan, 2010), 84.

38 Richard Soloway, *Demography and Degeneration: Eugenics and the Declining Birthrate in Twentieth-Century Britain* (Chapel Hill: The University of North Carolina Press, 1995), 80–1.

39 Galton cited in C. P. Blacker, *Eugenics: Galton and After* (London: Gerald Duckworth and Co., 1952), 86.

40 Galton cited in Marius Turda, ‘Race, Science, and Eugenics in the Twentieth Century’, in Bashford and Levine, eds, *The Oxford Handbook of the History of Eugenics*, 73.

41 John C. Waller, ‘Gentlemanly Men of Science: Sir Francis Galton and the Professionalization of the British Life-Sciences’, *Journal of the History of Biology*, 2001, 34, 96.

42 *Ibid*., 99 and 105.

43 Peter J. Bowler, *Reconciling Science and Religion: The Debate in Early-Twentieth-Century Britain* (Chicago: The University of Chicago Press, 2001), 70–2 and 150–3. Paul T. Phillips, ‘One World, One Faith: The Quest for Unity in Julian Huxley's Religion of Evolutionary Humanism’, *Journal of the History of Ideas*, 2007, 68, 613–33.

44 Julian Huxley, *Religion Without Revelation*, New and Revised Edition (London: Max Parrish, 1957), 1, 14, 25, 28.

45 James Moore, ‘R. A. Fisher: A Faith Fit for Eugenics’, *Studies in History and Philosophy of Biological and Biomedical Sciences*, 2007, 38, 120, 131.

46 Fisher, cited in Moore, ‘R. A. Fisher’, 121.

47 See Crackanthorpe in, *The Eugenics Review*, 1909, I (Apr.), 15.

48 EES Minutes, 31 March 1909, SA/Eug/L.1.

49 EES Minutes, Appendix II, 11 April 1951, SA/Eug/L.11.

50 Francis Galton, *Essays in Eugenics* (London: The Eugenics Education Society, 1909), 68.

51 Alfred Ploetz, along with Wilhelm Schallmayer, has been identified as a founder of the German eugenics movement. Sheila Faith Weiss, *Race Hygiene and National Efficiency: The Eugenics of Wilhelm Schallmayer* (Berkeley: University of California Press, 1987), 3, 99, and 149.

52 EES Minutes, 2 October 1911, SA/Eug/L.2.

53 *Ibid*.

54 Searle, for example has suggested that the subject of venereal disease was a distraction for the EES, see *Eugenics and Politics in Britain*, 14.

55 EES Minutes, 1 November 1911, SA/Eug/L.2.

56 EES Minutes, 14 February 1912, SA/Eug/L.2.

57 EES Minutes, 13 November 1917, SA/Eug/L.4.

58 *Ibid*.

59 EES Minutes, 17 April 1917, SA/Eug/L.4.

60 Susanne Klausen and Alison Bashford, ‘Fertility Control: Eugenics, Neo-Malthusianism, and Feminism’, in Bashford and Levine, eds, *The Oxford Handbook of the History of Eugenics*, 105.

61 G. K. Chesterton, *Eugenics and Other Evils* (London: Cassell and Company, 1922), 80. See the recent work, I. T. Ker, *G. K. Chesterton: A Biography* (Oxford: Oxford University Press, 2012).

62 See, for example, Letitia Fairfield, ‘The Case against Sterilization’ (1935) in Pauline M. H. Mazumdar, ed., *The Eugenics Movement: An International Perspective*, vol. III (Abingdon: Routledge, 2007).

63 Kevles, *In the Name of Eugenics*, 164–75; and Richard Soloway, ‘From Mainline to Reform Eugenics—Leonard Darwin and C P Blacker’ in Robert A. Peel, ed., *Essays in the History of Eugenics: Proceedings of a Conference Organised by the Galton Institute, London* (London: The Galton Institute, 1977).

64 Pauline Mazumdar, *Human Genetics and Human Failings: The Eugenics Society, its sources and critics in Britain* (London: Routledge, 1998), 96–130 and 142.

65 Lucy Bland and Lesley A. Hall, ‘Eugenics in Britain: The View from the Metropolis’, in Bashford and Levine, eds, *The Oxford Handbook of The History of Eugenics*, 216.

66 Evidence for this trend in the later period is found in 1935, for example, when the EES agreed to invite organisations ‘whose aims and activities touched upon eugenics’ to appoint delegates to the EES Council to help make the organisation more representative. EES Minutes, 12 March 1935, SA/Eug/L.10.

67 These figures do not include repeat visits, visits that were made to single groups such as church congregations, delegations sent to members of parliament, or meetings with other eugenics societies. The figure is thus a conservative illustration of meetings that were designed to help the EES meet with other organisations for the purpose of co-operation and the sharing of work or ideas. SA/Eug/L.1–9.

68 ‘Dr Drysdale’ was Alice Vickery, one of the first women to obtain a medical qualification. Vickery's partner and her son were both active in the Malthusian League and its campaign for access to birth control. She was critical of traditional sexual mores, and was active in the campaign for women's suffrage as well as the international birth control movement. Lesley A. Hall, ‘Vickery, Alice (1844–1929)’, *Oxford Dictionary of National Biography* (Oxford University Press, online edition, May 2010). EES Minutes, 26 January 1915, SA/Eug/L.4.

69 EES Minutes, 26 January 1915, SA/Eug/L.4.

70 David Renton, ‘Rivers, George Henry Lane Fox Pitt- (1890–1966)’, *Oxford Dictionary of National Biography* (Oxford University Press, online edition, May 2005).

71 George Pitt Rivers, ‘Memorandum on Policy and Other Matters’, 12 April 1932, SA/Eug/L.9.

72 EES Minutes, 12 March 1930, SA/Eug/L.9.

73 Rosen, *Preaching Eugenics*, see especially 111–37.

74 The Committee handed over its operations to the AES on 30 January 1926, prior to which several members already served in both organisations. American Philosophical Society Library, American Eugenics Society Records [Hereafter ‘AESP’] Box 7, Folder: AES Minutes, 1925–1926.

75 Letter from Henry Fairfield Osborn to Major Leonard Darwin, 5 December 1921. Henry Fairfield Osborn Papers, Archives of the American Museum of Natural History.

76 One of the names considered for the EES was ‘The Eugenics Education Society: For the Mental, Moral, & Physical Improvement of the Race’. EES Minutes, 25 November 1907, SA/Eug/L.1. Osborn cited in International Commission on Eugenics Ad Interim Committee of the United States of America, 9 June 1922, AESP Box 16.

77 Brian Regal, *Henry Fairfield Osborn: Race and the Search for the Origins of Man* (Aldershot: Ashgate, 2002), xii.

78 *Ibid*., 82.

79 David N. Livingstone, *Darwin's Forgotten Defenders: The Encounter between Evangelical Theology and Evolutionary Thought* (Michigan: William B. Eerdmans Publishing Company, 1987), 107–9.

80 Regal, *Henry Fairfield Osborn*, 39–40.

81 Letter from Osborn to Irving Fisher, 14 April 1922, Henry Fairfield Osborn Papers, Archives of the American Museum of Natural History.

82 Rosen, *Preaching Eugenics*, 115 and 117.

83 For the five clergymen, see ‘Financial Statement 1923’ on headed paper. AESP Box 5.

84 Kenneth Ludmerer, *Genetics and American Society: A Historical Appraisal* (Baltimore, MD: The Johns Hopkins University Press, 1972), 14.

85 AES Minutes, 24 February 1923, AESP, Box 5. On the AES budget see Rosen, *Preaching Eugenics*, 117.

86 *Report of the Committee on Formal Education of the American Eugenics Society Inc., 1928*, 6, AESP.

87 Robert W. Rydell, *World of Fairs: The Century-of-Progress Expositions* (Chicago: The University of Chicago Press, 1993), 40.

88 ‘Suggestions for Psychiatric Examiner’, AESP Box 11, AES Printing Orders, 1926–1942 #1.

89 AESP, Box 5 and Box 7, AES Minutes, 1927–1929.

90 Psalm xvi.6 ‘The lines are fallen unto me in pleasant places; yea, I have a goodly heritage’. AES Minutes, 31 October 1925, AESP Box 7.

91 AES Minutes, 28 March 1925, AESP Box 5.

92 The ‘catechism’ was discussed and promoted as a valuable tool in discussions found in AES Minutes for November 1926, January 1927, April 1931, December 1932, October 1934, and January 1935. AESP.

93 Unpublished autobiography of Leon Fradley Whitney, 186 (American Philosophical Society Library, Presented 1973).

94 For an account of Osborn's involvement in this Trial see Regal, *Henry Fairfield Osborn*, 160–2.

95 Edward J. Larson, *Summer for the Gods: The Scopes Trial and America's Continuing Debate over Science and Religion* (New York: Basic Books, 2006), 16 and 23.

96 *Ibid*., 27.

97 *Ibid*., 28. Barbara E. Ladner says that eugenics was the issue that pushed Bryan from the acceptance of a mild ‘creationist’ form of evolution towards an opposition to all forms of Darwinism. Ladner, ‘The Descent of Yoknapatawpha: Eugenics and the Origins of Faulkner's World’, in Susan Currell and Christina Cogdell, eds, *Popular Eugenics: National Efficiency and American Mass Culture in the 1930s* (Athens: Ohio University Press, 2006), 170. This view has been challenged in Adam R. Shapiro, *Trying Biology: The Scopes Trial, Textbooks, and the Antievolution Movement in American Schools* (Chicago: The University of Chicago Press, 2013), 50–2, 62–5.

98 On the subject of Bryan's interpretation of scripture, see Ronald L. Numbers, *The Creationists: From Scientific Creationism to Intelligent Design* (Cambridge, MA: Harvard University Press, 2006), 58. For his interaction with Osborn, see Regal, *Henry Fairfield Osborn*, 158–9.

99 *A Eugenics Catechism*, AESP Box 11, AES Printing Orders, 1926–1942 #4.

100 *Ibid*.

101 This reference to the gospel of Matthew (7:16) was popular with eugenicists. The EES planned to use this as the title of their propaganda film narrated by Julian Huxley. Minutes of the propaganda committee, 12 January 1937, SA/Eug/L.39.

102 Only two questions were given more space, one explaining the results of Mendel's pea experiments and the other describing how the ‘object of eugenics’ could be obtained.

103 *A Eugenics Catechism*.

104 Gowan Dawson, *Darwin, Literature and Victorian Respectability* (Cambridge: Cambridge University Press, 2007), 6 and 68; and Regal, *Henry Fairfield Osborn*, 170.

105 Weiss, *The Nazi Symbiosis*, 277.

106 *Eugenical News*, Vol. II, No. 12 (1917), 95.

107 *Eugenical News*, Vol. III, No. 6 (1918), 47–48; and *Eugenical News*, Vol. VIII, No. 8 (1923), 80.

108 Charles Benedict Davenport cited in Kevles, *In the Name of Eugenics*, 53.

109 See ‘Davenport, Charles Benedict’ in Ruth Clifford Engs, *The Eugenics Movement: An Encyclopedia* (Westport, CT: Greenwood Press, 2005), 40–1.

110 AES Minutes, 29 November 1927, AESP Box 7.

111 ‘Memorandum on the Eugenics Situation in the United States’, AESP Box 17.

112 Kevles, *In the Name of Eugenics*, 170.

113 Barry Alan Mehler, ‘A History of the American Eugenics Society, 1921–1940’ (PhD thesis, University of Illinois at Urbana-Champaign, 1988).

114 See especially: Mark A. Largent, *Breeding Contempt: The History of Coerced Sterilization in the United States* (New Brunswick, Rutgers University Press, 2008); and Currell and Cogdell, eds, *Popular Eugenics*.

115 AES Minutes, 4 May 1935, AESP Box 7, and Report of the Activities of the American Eugenics Society 1936–1937, presented at the 12th Annual Meeting of the AES, 23 September 1937. AESP Box 7.

116 A. E. Wiggam, ‘Conference of Publicists’, AESP Box 2.

117 *Ibid*.

118 ‘Preliminary Notes on the Conference on Eugenic Education’, 1937. AESP Box 2.

119 *Ibid*.

120 Sharon M. Leon, ‘“Hopelessly Entangled in Nordic Presuppositions”: Catholic Participation in the American Eugenics Society in the 1920s’, *Journal of the History of Medicine and Allied Sciences*, 2004, 59, 46. The AES presented a spirited critique of *Casti connubii* in *People* (April, 1934), 5–7.

121 Henry MacCracken, ‘Conference on Education and Eugenics’, 1937. AESP Box 2.

122 Matthew 16:25. There are similar quotations in the gospels of Mark and Luke.

123 ‘Moral Responsibilities of Parenthood’, 20 May 1936. AESP Box 17.

124 ‘American Eugenics Society Annual Meeting 5 May 1938’, AESP Box 17. This ‘limited use’ of sterilisation has recently been estimated at over 63,000 Americans coercively sterilised by the early 1960s, the majority in the 1930s when Osborn was speaking. Largent, *Breeding Contempt*, 1.

125 ‘American Eugenics Society Annual Meeting 5 May 1938’.

126 *Ibid*.

127 ‘Notes for Eugenic Program’, November 1934. AESP Box 17.

128 Letter from F. C. Schwartz to Frederick Osborn, AESP Box 17.

129 ‘Eugenics Credo’, AESP Box 17.

130 Jonathan Peter Spiro highlights this as the consensus of opinion throughout the period from the 1940s to the 2000s, and Diane Paul traces it to even earlier in his lifetime. Spiro, *Defending the Master Race: Conservation, Eugenics, and the Legacy of Madison Grant* (Burlington: University of Vermont Press, 2009), ix; Paul, *Controlling Human Heredity*, 17.

131 Letter from Grant to Henry Fairfield Osborn, 3 July 1930. Henry Fairfield Osborn Papers, Archives of the American Museum of Natural History.

